# Baseline symptom-related white matter tracts predict individualized treatment response to 12-week antipsychotic monotherapies in first-episode schizophrenia

**DOI:** 10.1038/s41398-023-02714-w

**Published:** 2024-01-13

**Authors:** Ying Chen, Shanming Liu, Bo Zhang, Gaofeng Zhao, Zhuoqiu Zhang, Shuiying Li, Haiming Li, Xin Yu, Hong Deng, Hengyi Cao

**Affiliations:** 1 Huaxi MR Research Center (HMRRC), Department of Radiology, West China Hospital of Sichuan University, Chengdu, China; 2 Hope Recovery and Rehabilitation Center, West China Hospital of Sichuan University, Chengdu, China; 3 Mental Health Center, West China Hospital of Sichuan University, Chengdu, China; 4 Shandong Daizhuang Hospital, Jining, Shangdong China; 5 Peking University Sixth Hospital, Peking University Institute of Mental Health, NHC Key Laboratory of Mental Health (Peking University), National Clinical Research Center for Mental Disorders (Peking University Sixth Hospital), Beijing, China; 6 Center for Psychiatric Neuroscience, Feinstein Institute for Medical Research, Manhasset, NY USA; 7 Division of Psychiatry Research, Zucker Hillside Hospital, Glen Oaks, NY USA

**Keywords:** Predictive markers, Schizophrenia

## Abstract

There is significant heterogeneity in individual responses to antipsychotic drugs, but there is no reliable predictor of antipsychotics response in first-episode psychosis. This study aimed to investigate whether psychotic symptom-related alterations in fractional anisotropy (FA) and mean diffusivity (MD) of white matter (WM) at the early stage of the disorder may aid in the individualized prediction of drug response. Sixty-eight first-episode patients underwent baseline structural MRI scans and were subsequently randomized to receive a single atypical antipsychotic throughout the first 12 weeks. Clinical symptoms were evaluated using the eight “core symptoms” selected from the Positive and Negative Syndrome Scale (PANSS-8). Follow-up assessments were conducted at the 4th, 8th, and 12th weeks by trained psychiatrists. LASSO regression model and cross-validation were conducted to examine the performance of baseline symptom-related alterations FA and MD of WM in the prediction of individualized treatment outcome. Fifty patients completed both clinical follow-up assessments by the 8th and 12th weeks. 30 patients were classified as responders, and 20 patients were classified as nonresponders. At baseline, the altered diffusion properties of fiber tracts in the anterior thalamic radiation, corticospinal tract, callosum forceps minor, longitudinal fasciculi (ILF), inferior frontal-occipital fasciculi (IFOF) and superior longitudinal fasciculus (SLF) were related to the severity of symptoms. These abnormal fiber tracts, especially the ILF, IFOF, and SLF, significantly predicted the response to antipsychotic treatment at the individual level (AUC = 0.828, *P* < 0.001). These findings demonstrate that early microstructural WM changes contribute to the pathophysiology of psychosis and may serve as meaningful individualized predictors of response to antipsychotics.

## Introduction

In schizophrenia, only ~70% of affected individuals respond to antipsychotic drug treatment, and even fewer go into remission [1, 2]. Pre-treatment prediction of the subsequent response could reduce the time spent on ineffective treatments, shorten patient suffering, and reduce possible mortality [3, 4]. The ability to identify brain biomarkers of antipsychotic nonresponders using magnetic resonance imaging may lead to improved prognosis and the detection of malleable central nervous system targets for the development of new treatment strategies.

Second-generation antipsychotics (SGAs) are widely believed to work by decreasing striatal dopamine via dopamine receptor blockage within the mesolimbic pathway to alleviate positive symptoms and increasing cortical dopamine via 5-HT(2A) antagonism in presynaptic neurons within the mesocortial pathway to improve negative symptoms [5, 6]. These neurotransmitters modulate synapses at glutamate (N-methyl-d-aspartate [NMDA]) receptors that are involved in synaptic plasticity and may cause delayed corollary discharges [7]. Animal studies noted that antipsychotic treatment following administration of the copper chelator cuprizone promoted oligodendrocyte development and remyelination [8, 9]. Thus, the integrity of WM may be a sensitive index of the brain pharmacological mechanism of action of SGA.

Diffusion tensor imaging (DTI) is widely used to evaluate the structural integrity of WM, and voxelwise metrics such as fractional anisotropy (FA) and mean diffusivity (MD) are generally considered sensitive measures for axonal/myelin damage [10–12]. A handful of DTI studies have documented the association between FA/MD of WM and response to treatment [13–15]. Further, inconsistencies prior work may be related to differences in clinical variables such as the illness course/chronicity, substance misuse, and previous exposure to antipsychotics. Therefore, investigating patients with drug-naive first-episode psychosis may address to disentangle which brain white matter changes may predict response to treatment.

To date, only three studies have investigated the effects of antipsychotic use on WM tracts in drug-naive first-episode schizophrenia. These studies show that antipsychotic medications appear to alter or improve FA or MD of WM, such as in the bilateral anterior cingulate gyrus (ACG), corticospinal tract (CT), anterior thalamic radiation (ATR), longitudinal fasciculi (ILF), inferior fronto-occipital fasciculi (IFOF), and uncinated fasciculus (UF) structural abnormalities, especially at remission [16–18]. However, while these studies typically report group-level WH tract differences between psychosis before and after antipsychotic administration, whether these findings have predictive value for individualized drug responses is unclear.

WH alterations may occur before the onset of psychosis, and FA/MD of WM changes have been reported in subjects with a high risk of psychosis [19, 20]. The severity of WM alterations such as fronto-temporal, fronto-occipital, and fronto-striatal WH at onset were associated with the severity and persistence of the signs and symptoms of schizophrenia, suggesting that baseline WM alterations may serve as an early marker for differentially characterizing patients with poor or good outcomes [21–23]. Furthermore, the cortex, especially the frontal lobe, is the core component of the dopamine projection system [24, 25]. However, it is still unknown whether psychotic symptom-related alterations in FA and MD of WM at the early stage of the disorder may provide aid to individualized prediction of drug response.

In this study, we investigated the above questions using DTI data acquired from first-episode schizophrenia patients with no prior medication. Patients underwent baseline structural MRI scans and were subsequently randomized to receive a single atypical antipsychotic throughout the first 12 weeks. It was hypothesized that the altered FA/MD of WM was related to the severity of psychotic symptoms at baseline, and those changes would show potential as individualized predictive biomarkers of response to SGA.

## Methods

### Participants

A total of 68 drug-naive patients between 18 and 45 years old were diagnosed with schizophrenia based on the Structured Clinical Interview for the DSM-IV Axis I disorder and had no previous psychiatric treatment. Patients underwent MRI scans and symptom ratings before assigned to a randomized open-label treatment with risperidone, olanzapine or aripiprazole for up to 1 year (Clinical trials.gov ID: NCT01057849). Clinical symptoms were evaluated using the eight “core symptoms” selected from the Positive and Negative Syndrome Scale (PANSS-8), which has more acceptable internal consistency and comparable sensitivity to early improvement in psychotic symptoms than the PANSS-30 [26]. This analysis included data only from the first 12 weeks of treatment. During this period, patients received a single antipsychotic that started with low dosage and gradually increased to a standard therapeutic range (3–6 mg risperidone, 15–30 mg aripiprazole, or 10–25 mg olanzapine per day) in 2 weeks. Follow-up assessments were conducted at the 4th, 8th, and 12th weeks by trained psychiatrists. To ensure the consistency and reliability of ratings across the study, three psychiatrists with more than 5 years of experience in clinical psychiatry attended a 1-week training workshop on the use of the rating instruments prior to the study. After training, they achieved an interrater reliability of 0.80 for the PANSS-8 score.

### Evaluation of treatment response

Treatment response was operationalized as a reduction in symptom severity to the levels required by the remission criteria of the Schizophrenia Working Group Consensus [27]. According to these criteria, clinical improvement is reached when a simultaneous rating of mild or less (equivalent to 1, 2, or 3) is given in all the following items of the PANSS-8: delusions (P1), conceptual disorganization (P2), hallucinatory behavior (P3), mannerisms and posturing (G5), unusual thought content (G9), blunted affect (N1), social withdrawal (N4), and lack of spontaneity and flow of conversation (N6). The clinical recommendation is that antipsychotic treatment with a specific drug should be continued for 6–8 weeks before switching to a different medication owing to lack of efficacy or adverse effects. Hence, in this study, we defined treatment response as meeting the remission criteria at the 8th or 12th week. Only Fifty patients completed both clinical follow-up assessments at the 8th and 12th weeks and were therefore included in this study as the final sample. The drop-out participants did not differ from the rest of the sample in characteristics and symptom severity (Supplementary Table S1).

### MRI data acquisition

The MRI scans were performed before medication using a GE Signa EXCITE 3.0-T scanner (GE Healthcare, Milwaukee, Wisconsin) equipped with an 8-channel phase array head coil. The DTI data were acquired using a bipolar diffusion-weighted spin‒echo planar imaging (EPI) sequence (TR = 10000 ms, TE = 70 ms) with a 128 × 128 matrix over a field of view of 240 × 240 mm and 42 axial slices of 3 mm thickness to cover the whole brain without gap. Each DTI dataset included 20 images of unique diffusion directions (B = 1000) and a nondiffusion image (B = 0). High-resolution T1 data were acquired using a 3D spoiled gradient (3D-SPGR) sequence: TR = 8.5 ms, TE = 3.5 ms, TI = 400 ms, flip angle = 12, 240*240 matrix over a field of view of 240*240 mm, and 156 axial slices of 1 mm thickness. All scans were reviewed by an experienced neuroradiologist to exclude gross brain abnormalities.

### Imaging processing

The routine DTI preprocessing included head motion and eddy current correction, brain extraction, and tensor model fitting was performed using FSL (FMRIB Software Library, http://www.fmrib.ox.ac.uk/fsl). We used automated fiber quantification software (AFQ) to identify 20 white matter traces in individual subjects. The identification procedure included three primary steps: whole-brain deterministic fiber tractography, waypoint ROI-based tract segmentation, and probability map-based fiber refinement using the 20-tract Johns Hopkins University white matter template. The 20 identified tracts were the left and right ATR, cingulum–cingulate (CC), cingulum–hippocampus pathway, inferior fronto-occipital fasciculus (IFOF), inferior longitudinal fasciculus (ILF), superior longitudinal fasciculus (SLF), uncinated fasciculus and arcuate fasciculus, and the forceps major of the splenium and the forceps minor of the genu of the corpus callosum. After tract identification, we smoothed each tract using a 10-point moving average filter to reduce local variation caused by imaging noise. The diffusion measurements along the tract core, defined as the tract profile, were extracted from each fiber tract, including the FA and MD values. Hence, each tract had two features, and each subject had 40 features to depict their global white matter status.

### Diffusion properties and clinical associations

To confirm the correlation between diffusion properties and symptoms at baseline, partial Pearson correlation was performed to examine relations between 40 white matter features and PANSS-8 scores at baseline, with age, gender, and duration of untreated psychosis as covariates. To adjust the significant values for multiple comparisons, we used the Benjamini–Hochberg false discovery rate (FDR *q* value selected to maintain the false positive error rate <0.05).

### Prediction of treatment outcome with diffusion properties

We next sought to investigate whether baseline FA and MD of WM would be capable of distinguishing antipsychotic responders from nonresponders at the individual level. To this end, we trained a cross-validated generalized LASSO regression model with treatment outcome as the dependent variable and baseline diffusion properties as predictors. To constrain the number of features in the model and meanwhile include all features relevant to the disorder, we preselected the FA and MD measures for model training. Here, only measures significantly associated with baseline symptoms at uncorrected *P* < 0.05 were included in the model as input predictors. We also trained the model with all baseline diffusion properties as predictors as a supplementary analysis (see Supplementary Materials).

The LASSO regression is an L1-norm regularization method that incorporates a shrinkage penalty term λ to avoid model overfitting, which coerces the coefficients of some less important predictors to be shrunken to zero. Specifically, the predictors included in the model were adjusted for age, sex, antipsychotic drug dosage, duration of untreated psychosis, and PANSS-8 scores at baseline. Similar to our prior work [28–30], a repeated nested cross-validation (CV) method (10 outer folds, each with 10 inter folds) was used in which the tuning parameter λ was optimized within the inner cycles and subsequently utilized to predict remaining subjects in the outer cycles. This procedure eventually yielded predicted probabilities of nonresponders for each individual in the main dataset, based on which the classification accuracy was calculated. To ensure the robustness of the results, we repeated the CV 100 times, each time by randomly parcellating the sample. The final classification performance was determined as the average area under curve (AUC) of the receiver operating characteristic (ROC) curves from the 100 runs, and the significance of the performance was determined by 1000 permutations. We also investigated whether the top features selected by the model (at least 8 out of 10 cycles) would be capable of predicting individualized symptom changes in first-episode schizophrenia as a supplementary analysis (see Supplementary Materials).

## Results

### 12-Week treatment outcome

By the end of the 12th week, 30 patients met the remission criteria as responders, and 20 patients were classified as nonresponders. Demographic and clinical variables at baseline were not significantly different between responders and nonresponders (Table 1).

**Table 1:** Clinical and demographic information for the first-episode schizophrenia patients.

Characteristic	Responders, *N* = 30	Nonresponders, *N* = 20	*t/F*	*P*
Age (years)	25.13 ± 7.45	25.65 ± 7.79	−0.24	0.815
Sex (M/F)	13/17	7/13	0.35	0.556
Duration of untreated psychosis (months)	5.73 ± 4.34	5.65 ± 4.63	0.07	0.949
Baseline PANSS-8	24.8 ± 5.85	23.65 ± 6.12	0.67	0.507
Antipsychotic type (risperidone/aripiprazole/olanzapine)	16/6/8	6/8/6	3.33	0.074
Mean antipsychotic dosage (olanzapine equivalents, mg/day)	19.08 ± 4.26	19.03 ± 4.87	0.34	0.564

### Diffusion properties and clinical associations at baseline

**Fig. 1:**
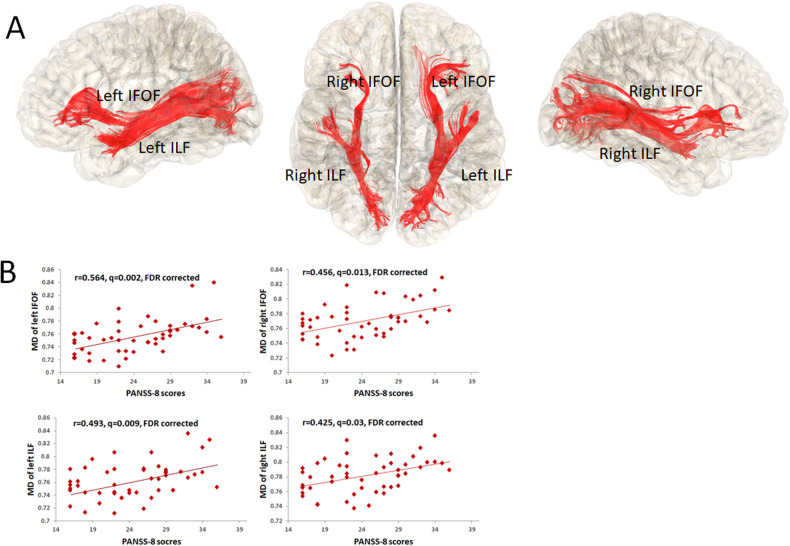
Relationship between diffusion properties and the severity of core symptoms at baseline in first-episode schizophrenia. **A** Fiber tracts significantly associated with symptoms. **B** The red lines indicate MD of fiber tracts were positively correlated with PANSS-8 scores. IFOF inferior fronto-occipital fasciculi, ILF longitudinal fasciculi, MD mean diffusivity.

In partial correlation analysis between PANSS-8 scores and FA/MD of each fiber tract, we found positive correlations between PANSS-8 scores and average MD of the following fiber tracts after FDR correction: left and right IFOF (*r* = 0.564, *q* = 0.002; *r* = 0.456, *q* = 0.013), and left and right ILF (*r* = 0.493, *q* = 0.009; *r* = 0.425, *q* = 0.03) (Fig. 1). At a more liberal threshold without FDR correction, PANSS-8 score was significantly correlated with the FAs of the left ILF (*r* = -0.296, *P* = 0.044) and right ILF (*r* = −0.374, *P* = 0.01), as well as MDs of the left ATR (*r* = 0.358, *P* = 0.013), left corticospinal (*r* = 0.362, *P* = 0.012), right corticospinal (*r* = 0.389, *P* = 0.007), genu of corpus callosum (*r* = 0.346, *P* = 0.017), and right SLF (*r* = 0.376, *P* = 0.009). Therefore, these eleven measures (two FA measures and nine MD measures) were subsequently used as predictors in the LASSO regression model.

### Classification performance of treatment response

**Fig. 2:**
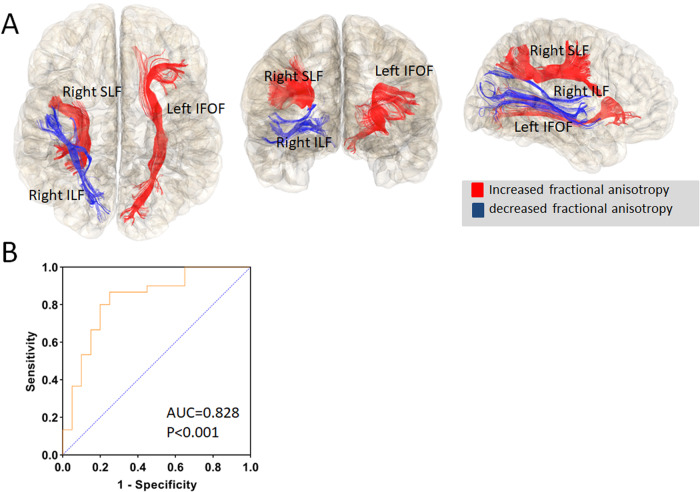
Diffusion properties in discriminating nonresponders from responders in first-episode schizophrenia after 12-week antipsychotic monotherapies. **A** The selected diffusion properties showing highest predictability for nonresponders from cross-validated LASSO regression in first-episode schizophrenia. Red and blue indicated increased and decreased fractional anisotropy, respectively. **B** The receiver operating characteristic (ROC) curve for nonresponders. ILF longitudinal fasciculi, IFOF inferior fronto-occipital fasciculi, SLF superior longitudinal fasciculus.

The average AUC from the 100 repeats of the LASSO regression model was 0.828 (range: 0.81–0.86) (*P* < 0.001, average sensitivity = 0.867 and average specificity = 0.636). Here, the FA of the right ILF and MDs of the left IFOF and the right SLF had nonzero coefficients at least 8 out of 10 cycles during all 100 repeats, and were therefore selected as final features. The post-hoc *t* test revealed significantly higher FA of the right ILF (*t* = 5.69, *P* < 0.001) but lower MDs of the left IFOF and the right SLF in responders compared with nonresponders (*t* = −2.25, *P* = 0.029; *t* = −2.62, *P* = 0.012) (Table 2 and Fig. 2).

**Table 2:** Significant features for discriminating treatment responders and nonresponders.

Selection times	Hemisphere	Label	Feature type	Responders	Nonresponders	*t*	*P*
10	Right	ILF	FA	0.45 ± 0.2	0.42 ± 0.02	5.69	<0.001
9	Left	IFOF	MD	0.75 ± 0.02	0.77 ± 0.03	−2.25	0.029
8	Right	SLF	MD	0.68 ± 0.02	0.7 ± 0.03	−2.62	0.012

*ILF* longitudinal fasciculi, *IFOF* inferior fronto-occipital fasciculi, *SLF* superior longitudinal fasciculus, *FA* fractional anisotropy, *MD* mean diffusivity.

## Discussion

Here, we provide evidence for a baseline psychotic symptom-related white matter tract biomarker that potentially predicts response to antipsychotic treatment in first-episode schizophrenia. Importantly, the study sample was treatment-naive to ensure that the findings were not confounded by the drug. Two main findings emerged from this study. First, the altered diffusion properties (FA or MD) of fiber tracts in the ATR, corticospinal tract, callosum forceps minor, IFOF, ILF and SLF were related to the severity of symptoms, demonstrating that early microstructural WH changes contribute to the pathophysiology of psychosis. Second, these abnormal fiber tracts, especially the ILF, IFOF, and SLF, significantly predicted the response to antipsychotic treatment at the individual level. This suggests that these symptom-related WH changes could be an outcome marker after the onset of psychosis or even a target for intervention and preventive strategies.

Our findings decreased FA and increased MD of several fiber bundles throughout the brain correlated with core positive and negative symptom severities consistent with a “disconnection” hypothesis of symptoms in schizophrenia [31, 32]. Several meta-analytic studies have investigated the role of WH irregularities in schizophrenia spectrum disorders [33–36]. A meta-analysis of WM alteration in patients with FES indicated widespread abnormalities across white matter tracts, with evidence for reductions in FA in the corpus callosum, the left ILF and IFOF [34]. Even in chronic schizophrenia, the meta-analysis of 15 DTI studies also observed significant FA reductions in the genu and splenium of corpus callosum, the left anterior thalamic radiation, the left IFOF and ILF [36]. Moreover, the relationship between aberrant FA of these WM tracts and psychotic symptoms of schizophrenia were reported among previous studies [22, 37]. Consistent with this study, several studies found the inverse relationship between FA of SLF, ILF, and IFOF and negative symptoms and auditory verbal hallucinations in schizophrenia [37–39]. Taken together, these implied that white matter dysintegrity may represent a “trait” marker, related to the underlying pathophysiology in schizophrenia.

Beyond the group level for white matter correlated with symptoms at baseline, our longitudinal follow-up study also provided evidence that these baseline psychotic symptom-related FA and MD of WM may serve as an individualized predictor for antipsychotic treatment response in patients with schizophrenia. Similar to our findings, previous group-level analysis studies found more widespread FA decreases at baseline in FEP patients with a subsequent poorer response, and that baseline global white matter network organization showed greater alterations in FEP patients who subsequently showed a poorer treatment response [40–42]. Taken together, all these studies highlight the usefulness of baseline WM integrity in predicting response to treatment. Furthermore, the LASSO regression model in this study correctly classified 82.8% of patients as responsive, which may represent an important preliminary step to provide clinicians with decision support in selecting the ideal antipsychotic treatment for schizophrenia in a personalized manner. Further studies using larger and independent samples are required to replicate these findings.

We focused on predicting biomarkers of symptom-related FA and MD in WM. Longitudinal studies have observed that longitudinal increases in FA values, especially in the IFOF, ILF, SLF, and anterior thalamic radiation, are significantly correlated with improved symptoms at follow-up [17, 18, 43]. Consistent with these findings, the top treatment response predicting features in our study were located in the ILF, IFOF, and SLF. These WM tracts connect frontal, temporal, parietal and occipital areas, which have been implicated in several cognitive functions, such as visuospatial processing, emotional regulation, memory and language, in schizophrenia [44, 45]. In addition, altered FAs in the SLF and IFOF have been found to be a biomarker for auditory hallucinations, and FA in the ILF has been linked to positive symptoms [46, 47]. Furthermore, these regions are major target of dopamine signaling. Evidence from animal models suggested that the upregulation of D2 receptors in the frontal, parietal, temporal and occipital lobes and the downregulation of D1 receptors in the prefrontal and temporal cortices may be an important component of the therapeutic response to neuroleptic drugs [48]. These receptor blockades have been shown to promote oligodendrocyte repopulation and remyelination of experimentally demyelinated cells in mice [49]. We also found that nonresponders had more severe WM damage as lower FA and higher MD in these fiber bundles at baseline than responders. These findings together suggest that the alterations in the IFOF, ILF and SLF may represent neural markers of the severity and persistence of the signs and symptoms of schizophrenia, and may compromise the potential effects of antipsychotics.

This study has some limitations. First, participants in our study were randomized to receive a single standardized treatment with one of three antipsychotics, but the sample was too small to rule out changes in response patterns based on different medications. Future investigations are encouraged to provide a comparison of the effectiveness of different drugs. Second, the study did not include a placebo control group, so a potential placebo or time effect cannot be excluded. Due to ethical issues, these effects are normally nested in clinical studies and cannot be completely removed. Third, as a machine learning study focusing on individual prediction, the sample size in this study was relatively small, and it lack an independent validation sample. Therefore, these findings should be externally verified with larger samples in the future.

In conclusion, altered FA and MD of fiber tracts in the ATR, corticospinal tract, callosum forceps minor, IFOF, ILF, and SLF were related to the severity of symptoms in first-episode schizophrenia. These effects on WM tracts are not influenced by pharmacotherapy and therefore appear to be disease-related. These baseline psychotic symptom-related WM tracts, especially ILF, IFOF, and SLF, may serve as meaningful individualized predictors of response to SGA. These results may represent an important first step of the translational value of baseline brain structural measures in precision psychiatry.

### Supplementary information

Supplement materials

## Data Availability

The data that support the findings of this study are available from the corresponding author upon reasonable request.
