# Supplementary material for: Baseline symptom-related white matter tracts predict individualized treatment response to 12-week antipsychotic monotherapies in first-episode schizophrenia
Source: Transl Psychiatry. 2024 Jan 13;14:23. doi: 10.1038/s41398-023-02714-w (PMC10787827; doi:10.1038/s41398-023-02714-w)
Supplement: Supplementary file 1 — Supplement materials [file 41398_2023_2714_MOESM1_ESM.docx]

Supplement materials

Table S1 Clinical and demographic information for excludes subjects and includes subjects

| Characteristic | Excluded subjects  N=18 | Included subjects  N=50 | t/F | P |
| --- | --- | --- | --- | --- |
| Age(years) | 24.78±7.89 | 25.33±7.48 | 0.268 | 0.79 |
| Sex(M/F) | 7/11 | 20/30 | 0.007 | 0.934 |
| Duration of untreated psychosis (months) | 6.67±4.38 | 5.76±4.55 | -0.799 | 0.427 |
| Baseline PANSS-8 | 24.83±4.27 | 24.34±5.93 | -0.323 | 0.747 |

Prediction of Treatment Outcome with All Diffusion Properties

We investigated the potential of all baseline fractional anisotropy (FA) and mean diffusivity (MD) values of white matter in distinguishing antipsychotic responders from non-responders on an individual basis. A cross-validated generalized LASSO regression model was trained, employing treatment outcome as the dependent variable and incorporating all baseline diffusion properties as predictors. These predictors were adjusted for age, sex, antipsychotic drug dosage, duration of untreated psychosis, and PANSS-8 scores at baseline, following similar methodology to our previous work. Utilizing a repeated nested cross-validation approach (10 outer folds, each with 10 inner folds), we optimized the tuning parameter λ within the inner cycles, subsequently applying it to predict the remaining subjects in the outer cycles. This process yielded predicted probabilities of non-responders for each individual in the main dataset, from which classification accuracy was determined.

We obtained an AUC of 0.825, sensitivity of 0.866, and specificity of 0.55 (p<0.05). Notably, fractional anisotropy (FA) values of the left inferior fronto-occipital fasciculus (IFOF), and the right inferior longitudinal fasciculus (ILF), along with mean diffusivity (MD) values of the left IFOF and the right superior longitudinal fasciculus (SLF) demonstrated non-zero coefficients in at least 5 out of 10 cycles, thus, were selected as final features. Notably, these selected features align closely with those anticipated from symptom-associated predictors.

Prediction of Individualized Treatment Responses

To estimate individualized treatment responses, we employed a linear mixed model considering time points (baseline, 4, 8, and 12 weeks) as fixed variables, with random slopes and intercepts estimated for each individual. This model was applied to quantify subject-specific slope coefficients for PANSS-8 scores over time, reflecting individualized average change rates across the follow-up period for clinical symptoms.

Subsequently, we investigated whether the FA of the right ILF and MDs of the left IFOF and right SLF could predict individualized clinical outcomes in first-episode schizophrenia. For this purpose, we utilized the LASSO regression model combined with cross-validation. The resulting individualized change rate for PANSS-8 scores was included in LASSO regression models as the dependent variable (DV), with baseline the FA of the right ILF and MDs of the left IFOF and right SLF serving as predictors.

The cross-validated generalized LASSO regression model revealed significant correlations between the predicted slopes and the observed slopes (r=0.271, p<0.05).
